# Unveiling the Therapeutic Promise of Epigenetics in Vascular Cognitive Impairment and Vascular Dementia

**DOI:** 10.14336/AD.2025.0010

**Published:** 2026-02-05

**Authors:** Sharmelee Selvaraji, Jasmine Mosberger, David Y. Fann, Mitchell KP. Lai, Christopher Li Hsian Chen, Thiruma V. Arumugam

**Affiliations:** ^1^Memory Aging and Cognition Centre, Department of Pharmacology, Yong Loo Lin School of Medicine, National University of Singapore, Singapore.; ^2^Integrative Sciences and Engineering Programme, NUS Graduate School, National University of Singapore.; ^3^Research Laboratory of Electronics, Department of Materials Science and Engineering, Massachusetts Institute of Technology, Cambridge, MA 02139, United States of America.; ^4^Department of Pharmacology, Yong Loo Lin School of Medicine, National University of Singapore, Singapore.; ^5^Healthy Longevity Translational Research Program, Yong Loo Lin School of Medicine, National University of Singapore, Singapore.; ^6^Centre for Healthy Longevity, National University Health System (NUHS), Singapore.; ^7^Department of Psychological Medicine, Yong Loo Lin School of Medicine, National University of Singapore, Singapore.; ^8^School of Pharmacy, Sungkyunkwan University, Suwon, Republic of Korea.; ^9^La Trobe Institute for Molecular Science, La Trobe University, Melbourne, Australia.; ^10^Department of Microbiology, Anatomy, Physiology and Pharmacology, School of Agriculture, Biomedicine and Environment, La Trobe University, Melbourne, Australia.

**Keywords:** Epigenetics, DNA Methylation, Histone Modification, Non-Coding RNA, Gene Expression, Environment, Vascular Dementia, Vascular Cognitive Impairment

## Abstract

Vascular dementia (VaD) is a progressive neurodegenerative disease characterized by cognitive decline and memory deficits. Despite its significant prevalence and impact, the pathophysiology of VaD remains poorly understood, and current treatments are limited to symptom management. Emerging evidence highlights the importance of lifestyle-associated risk factors in VaD, emphasizing the role of gene-environment interactions, particularly in the realm of epigenetics. While preclinical studies using animal models have provided valuable insights into epigenetic mechanisms, the translatability of these findings to human clinical settings remains limited, and research into VaD-specific epigenetics is still in its infancy. This review aims to elucidate the intricate interplay between epigenetics and VaD, shedding light on potential therapeutic interventions rooted in epigenetic mechanisms. By synthesizing insights from existing literature, we also discuss the challenges and opportunities in translating preclinical findings into clinically viable treatments, underscoring the need for further research to bridge the gap between animal models and human applications.

## Introduction

Dementia is increasingly considered an epidemic of forgetfulness with a new case being diagnosed every three seconds worldwide [1]. Used as an umbrella term, dementia describes a group of symptoms associated with a decline in cognitive function [2]. These symptoms include loss of memory, impaired language and construction skills that are often accompanied by delusions and agitations [3]. Affecting over 50 million people worldwide, the prevalence of dementia is expected to triple by 2050 [1]. This vast increase is attributed to an aging population where age is a high-risk factor for dementia. Apart from a healthcare burden, the associated socio-economic burden is rising as well, making dementia an urgent problem. In fact, the World Health Organisation (WHO) has since recognised and declared dementia to be a public health priority [4].

Vascular dementia (VaD) is a progressive neurodegenerative disease that leads to cognitive decline and memory deficits [5]. It is characterised mainly by reduced cerebral blood flow (CBF), also known as chronic cerebral hypoperfusion (CCH), the presence of white matter hyperintensities and increased blood-brain barrier (BBB) permeability. Clinical features of VaD include mood disorders, psychomotor loss, and impairments in memory and executive functioning [5]. VaD contributes to approximately 20% of dementia cases [5]. Despite the number of cases being proportionately less, VaD has a rapid stepwise decline in disease progression as opposed to a gradual decline in Alzheimer’s disease (AD) which is the most common cause of dementia [1]. This thus contributes to a higher mortality rate among VaD patients [6]. With a rapidly aging population, the number of VaD cases and the associated mortality rate are actively on the rise [7]. To further aggravate the problem, VaD poses diagnostic challenges. It is often deduced by an elimination method, filtering from the diagnosis of other dementia patients, which is usually based upon identified risk factors and medical history of vascular conditions [8]. In a clinical setting, a comprehensive evaluation is conducted on individuals using the Diagnostic and Statistical Manual of Mental Disorders IV (DSM-IV) criteria to systematically diagnose VaD patients [9,10].

Epidemiologically, sporadic cases of VaD are more prevalent than familial ones which are estimated to be less than 1% [11]. Since aging is the primary risk factor for VaD, age-associated conditions and diseases become potential risk factors. The role of vascular risk factors as a major driver of VaD is rather pertinent. These include hypertension, diabetes mellitus and hyperlipidemia [5]. For example, diabetes mellitus has been known to double the risk of dementia and poses the risk of worsening a patient’s condition from mild cognitive impairment to dementia [5]. Lifestyle factors such as smoking, alcohol consumption, physical inactivity and poor diet have also been demonstrated to contribute to the risk of VaD [12]. These environmental risk factors often co-occur in VaD patients thus complicating the identification and treatment of the disease. Moreover, given that the pathophysiology of VaD is not well understood, it is not surprising that current treatments remain purely symptomatic and in certain cases focused on stroke prevention instead due to the increased risk of post-stroke vascular dementia [13]. Therefore, it is strategic to focus on early intervention to facilitate preventive treatment options.

**Figure 1.**
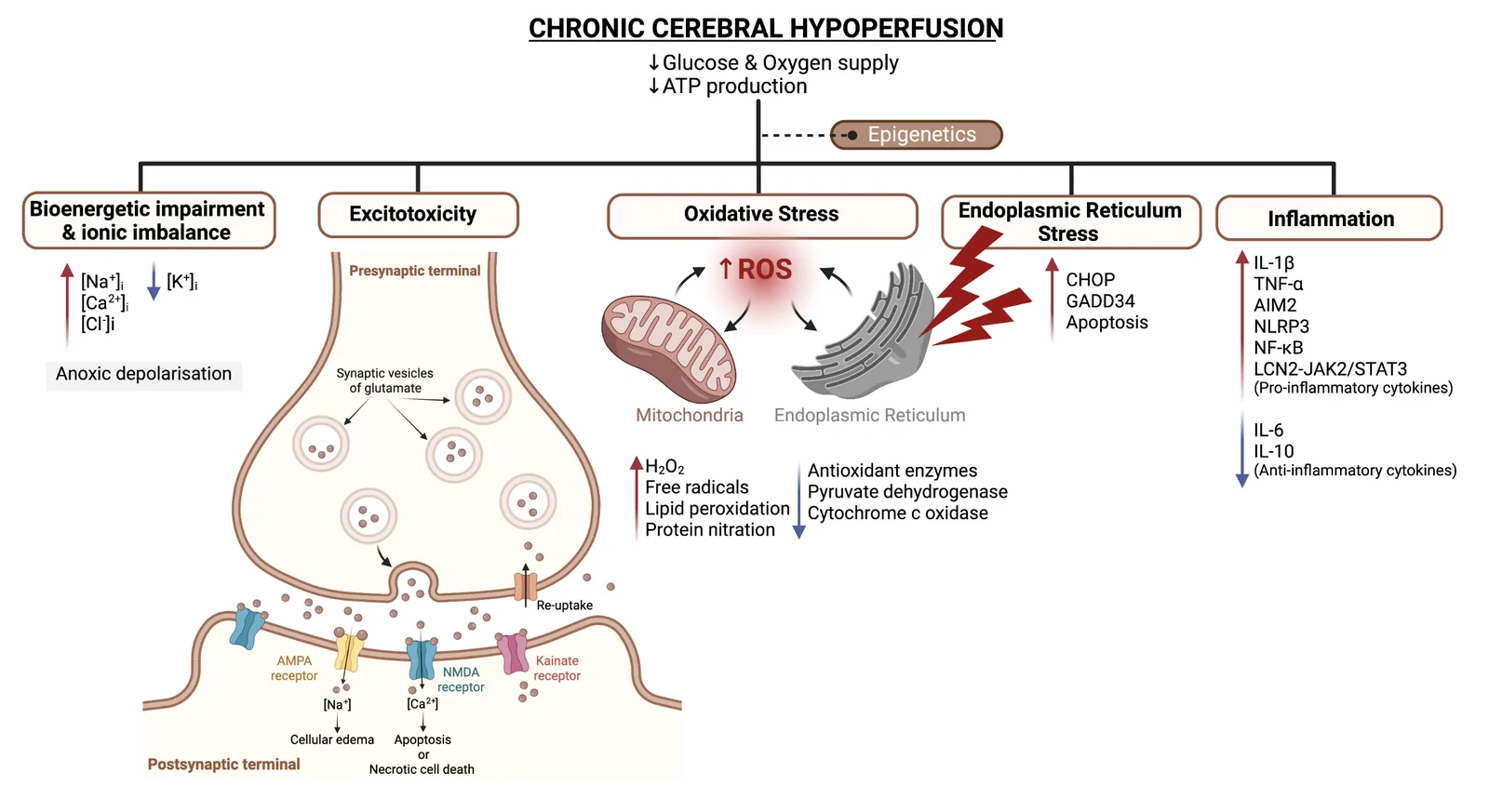
**Schematic diagram summarising the pathological mechanisms of CCH**. The reduced glucose and oxygen supply results in reduced ATP production and hence initiating a cascade of interlinked molecular events as shown in the illustration. Bioenergetic impairment and ionic imbalance involves increased intracellular levels of Na^+^, Ca^2+^ and Cl^-^ ions and reduced intracellular levels of K^+^ ions. The subscript ‘i’ indicates the intracellular concentration. This ionic imbalance results in the neurons to undergo anoxic depolarisation. The overactivation of excitatory amino acid receptors, glutamate receptors such as N-methyl-D-aspartate receptor (NMDA receptor), α-amino-3-hydroxyl-5-methyl-4-isoxazole-propionate receptor (AMPA receptor) and kainate receptor which result in excitotoxicity is shown figuratively. The differential consequences of increased [Na^+^] resulting in cellular edema and increased [Ca^2+^] resulting in apoptosis or necrotic cell death have been highlighted. Increased reactive oxygen species (ROS) levels from the primary sources mitochondria and endoplasmic reticulum (ER) have been shown to increase ROS species and reduce levels of antioxidant enzymes. ER stress has been shown to increase ER-stress related genes such as CCAAT-enhancer-binding protein homologous protein (CHOP) and growth-arrest- and DNA-damage-inducible gene 34 (GADD34) alongside apoptosis. Inflammatory response in CCH has been represented with increasing levels of pro-inflammatory cytokines and decreasing levels of anti-inflammatory cytokines. Epigenetics is represented at the point of divergence from CCH to the respective pathological mechanisms. This is to indicate the postulation that epigenetics is an upstream pathological mechanism that modulates the altered gene expressions observed downstream.

Emerging evidence have demonstrated a significant connection between environmental factors and the pathogenesis of VaD. In particular, the altered gene expression observed in VaD may serve as a diagnostic biomarker of the disease, although the modulatory mechanisms remain unknown. Overall, while there are tests and measures conducted to diagnose VaD patients and treat them for their symptoms, VaD still carries much controversy with disagreement regarding the validity of the clinical criteria [14,15]. Therefore, to be able to identify, diagnose and treat VaD patients, the gap between the knowledge of clinical manifestation and the underlying molecular mechanism of the disease has to be bridged. Several postulations have been made with regards to disease pathogenesis. CCH leads to a diminished supply of essential nutrients, notably glucose and oxygen, to the brain. This deprivation instigates a cascade of molecular phenomena, encompassing bioenergetic dysregulation, ionic perturbations, excitotoxicity, oxidative stress, endoplasmic reticulum stress, and inflammatory responses (Fig. 1). As a result, CCH emerges as a significant pathological characteristic within the spectrum of vascular cognitive impairment (VCI) and VaD. In the subsequent section, we delve into the principal pathological mechanisms triggered by CCH, including bioenergetic impairment, excitotoxicity, oxidative stress, endoplasmic reticulum stress, and neuroinflammation. We also shed light on their intricate interplay and implications.

## Pathological Mechanisms of Chronic Cerebral Hypoperfusion (CCH)

### Bioenergetic Impairment and Ionic Imbalance

Bioenergetics is fundamental to the flow and transformation of energy within living organisms. This generation of energy is facilitated by individual biochemical cellular pathways such as glycolysis, the Krebs cycle and oxidative phosphorylation [16]. Glucose, the main source of energy for the brain is metabolised by cellular glycolysis and majorly by mitochondrial oxidative phosphorylation to produce adenosine triphosphate molecules (ATP) [17]. The flow of electrons through the electron transport chain in the mitochondria allows proton pumps to harness energy to oxygen. The proton gradient generated is then coupled to the synthesis of ATP by the ATP synthase complex [18]. Bioenergetic impairment occurs when there is a disruption to the cellular energy metabolism. The decreased energy supply results in reduced ATP production which then impairs the ATP-dependent sodium-potassium pump (Na^+^/K^+^ ATP synthase), causing ionic imbalance [19].

When blood flow to the brain is reduced, the delivery of both glucose and oxygen is also reduced. Consequently, this results in the production of energy in the form of ATP to be decreased which then causes mitochondrial ATP synthase to run in reverse, depleting cellular ATP stores [20]. The reduction in ATP levels thus results in ionic imbalance through reduced activity of the Na^+^/K^+^ ATP synthase pump causing the resting membrane potential to increase allowing voltage-gated ionic channels to open, inducing an influx of Na^+^, Ca^2+^ and Cl^-^ ions and efflux of K^+^ ions across the plasma membrane. This ionic imbalance results in the neurons undergoing anoxic depolarisation, a phenomenon observed under ischemic conditions [21]. Defects in mitochondrial bioenergetics is a key cause contributing to the pathogenic state of VaD [22]. The disruption of brain energy metabolism in a rat model of CCH was confirmed through various biochemical assessments of oxidative phosphorylation parameters, including the respiration control index, oxidative phosphorylation coefficient, basal oxygen uptake rate, ADP-stimulated oxygen uptake rate, and oxidative phosphorylation rate [23]. In a mouse model of VaD, mitochondrial bioenergetic deficits were observed in the hippocampi, characterized by reduced expression and activity of complex IV in the electron transport chain [24]. Attempts have even been made to mitigate bioenergetic impairment as a form of therapeutic intervention for CCH in VaD. This includes studies involving use of natural flavonoid molecules such as pinocembrin and baicalein with antioxidant and superior free radical scavenging properties and supplementation of nicotinamide adenine dinucleotide (NAD^+^), a coenzyme I, to ameliorate mitochondrial damage and decrease reactive oxidative species (ROS) production [25,26,27].

### Excitotoxicity

Bioenergetic impairment during CCH results in overactivation of excitatory amino acid receptors. This, in turn, triggers excitotoxicity which is a complex process that causes neuronal death [28]. Activation of glutamate excitatory receptors, including N-methyl-D-aspartate receptors (NMDARs), α-amino-3-hydroxy-5-methyl-4-isoxazolepropionic acid receptors (AMPARs), and kainate receptors, leads to a significant influx of sodium (Na+) and calcium (Ca2^+^) ions into neurons. The increased influx of Na^+^ can result in an osmotic influx of water molecules causing neuronal swelling, but it is often reversible [29]. However, glutamate-stimulated Ca^2+^ influx through NMDARs and voltage-gated calcium channels often results in irreversible excitotoxic injury causing neuronal cell death [30]. The abnormally high levels of intracellular Ca^2+^ also activates degradative enzymes, generates toxic free radicals and disrupts cellular energy production due to oxidative stress [31].

The extent of damage it incurs depends on the extent of severity of CCH in VaD. The role of excitotoxicity in driving the pathogenesis of VaD has been shown and hence have been strategically targeted as a form of potential therapeutic intervention [32]. This includes memantine, an antagonist of NMDA-glutamate receptors [33,34] and Activin A, an exocrine protein of activity-regulated inhibitor of death which promotes NMDAR phosphorylation that regulates Ca^2+^ influx [35].

### Oxidative Stress

Oxidative stress refers to the imbalance between endogenous oxidants and antioxidants, resulting in the rise of ROS such as free radicals (e.g. superoxide anions, hypochlorite, peroxides), hydroxyl radical (most reactive oxygen species) and non-radicals (hydrogen peroxides) [36]. These ROS are generated by various sources which include the plasma membrane, cytosol, peroxisomes, endoplasmic reticulum and the membranes of mitochondria [37]. Given the high metabolic activity with high oxygen consumption and lipid-rich content, it is unsurprising that the brain is highly susceptible to oxidative stress [38,39,40]. The overproduction and impaired clearance of free radicals both contribute to an increase in cytosolic ROS levels. Under reduced cerebral blood flow, the mitochondrial dysfunction results in energy metabolism failure which increases ROS production [41]. Excessive ROS, in turn, increases lipid peroxidation and protein nitration, decreases the activity of antioxidant enzymes, and thus induces oxidative stress [42]. ROS, under physiological levels, play a key role as cerebral vasoconstrictors and vasodilators thus regulating brain perfusion [43]. Therefore, ROS, which impact vascular tone, could potentially exacerbate the reduction in cerebral blood flow [42]. Essentially, cerebral hypoperfusion can trigger oxidative stress, and this vascular oxidative stress, in turn, has a deleterious effect on cerebral hypoperfusion, thus perpetuating a vicious cycle of injury.

VaD patients were found to have increased systemic levels of oxidative stress [44]. High levels of peripheral oxidative stress markers and reduced antioxidant functions were observed in VaD patients [36]. In addition, another study identified a decline in pyruvate dehydrogenase protein levels and cytochrome c oxidase, which was associated with increased oxidative stress and decreased cellular respiration respectively in VaD [24]. Physiologically, the system responds to increased oxidative stress under ischemic insult. This has been studied in CCH-induced neurodegenerative rats where the antioxidant enzymes glutathione peroxidase, superoxide dismutase and catalase were observed to be increased [45]. However, the damage often supersedes the compensatory response, thus emphasising the need for external administration of antioxidants to slow it down, if not to inhibit, the cognitive impairment induced by CCH. Studies on rat models of CCH have shown that curcumin protects the brain from oxidative stress by increasing the expression of uncoupling protein 2 that dissipates the proton gradient thereby lowering the mitochondrial membrane potential [46] and green tea polyphenols have shown improvement in spatial cognitive abilities by potentially modulating oxidative stress [47]. In addition, acetylcholinesterase inhibitors and statins have also been explored to mitigate oxidative stress-induced vascular dysfunction [48].

### Endoplasmic Reticulum Stress

The endoplasmic reticulum (ER) performs numerous critical functions, including protein synthesis and transport, calcium storage, and lipid metabolism (Schwarz & Blower, 2016). It plays a vital role in the synthesis and post-translational modifications of secretory molecules, crucial for maintaining calcium (Ca2^+^) homeostasis [49]. As the site of translation, protein folding, and transport, any disruption to its physiological functions—such as ER-calcium depletion, hypoxia, or oxidative stress—can lead to protein misfolding and the accumulation of unfolded proteins. These stressors activate an adaptive stress response known as the unfolded protein response (UPR), an integrated signal transduction pathway. This pathway involves three ER membrane-associated sensors: protein kinase R-like endoplasmic reticulum kinase (PERK), inositol-requiring enzyme 1 (IRE1), and activating transcription factor 6 (ATF6) [49].

Under prolonged ER stress, sustaining cellular proteostasis is not feasible, thus resulting in accumulation of misfolded proteins and activation of terminal UPR [50]. In neurodegenerative diseases, terminal UPR mediates synaptic dysfunction, neuronal cell death and axonal degeneration. For instance, the presence of ER stress was reported in post-mortem brain samples of AD patients with specific upregulation of phosphorylated IRE1 (p-IRE1) and p-PERK levels in AD neurons [49]. In the context of vascular diseases, ER stress has been found to play a role in the mechanisms underlying ischemia/reperfusion neuronal damage. Attenuation of ER stress-induced apoptosis has been found to confer protection against ischemia and reperfusion injury [51]. ER stress is increasingly recognized as a contributing factor in the development of vascular cognitive impairment (VCI) [52]. Research using neuronal models of CCH has demonstrated that zinc-induced neurotoxicity plays a role in its pathogenesis, leading to the upregulation of ER stress-related genes, such as CCAAT-enhancer-binding protein homologous protein (CHOP) and growth-arrest- and DNA-damage-inducible gene 34 (GADD34) [53]. Furthermore, the ER stress pathway has been implicated in zinc-induced neurotoxicity, suggesting it may act both as a cause and a consequence in the progression of VaD [54].

### Neuroinflammation

Inflammation is a complex biological response to stimulation by invading pathogens or endogenous signals of danger or repair. Neuroinflammation, involving the inflammatory response in the brain and spinal cord, is mediated by the production of pro-inflammatory cytokines (e.g. IL-1β, IL-6, TNF), chemokines, ROS and other secondary mediators [55]. The innate and adaptive immune responses in the central nervous system are initiated in neurodegenerative diseases. Microglia, astrocytes and endothelial cells tend to act as antigen-presenting cells while neurons may secrete complement factors, chemokines and danger-associated molecular patterns thus activating the immune response [56]. VaD patients have been observed to have increased inflammatory biomarkers both in their plasma and cerebrospinal fluids which is associated with cognitive decline [57]. The expression of proinflammatory cytokines significantly increases under CCH triggering a cascade of events leading to brain tissue damage which include generation of free radicals, obstruction of microvessels and secretion of cytotoxic enzymes [41]. Studies have shown that CCH specifically activates the AIM2 and NLRP3 inflammasomes in glial cells [58,59]. Transient receptor potential melastatin 2 (TRPM2), a Ca^2+^ permeable channel functionally expressed in the brain, plays a pivotal role in mediating chronic inflammation during CCH via activation of the microglia [60]. Moreover, the microvascular changes that occur during CCH causes continuous oligodendrocyte death that reduces the synthesis of myelin fibres resulting in its degeneration that, in turn, increases low-grade inflammation [61].

The increased inflammatory response and the associated increased white matter lesions, blood-brain barrier damage and worsening of cerebral hypoperfusion have thus led to neuroinflammation being targeted as a potential pharmacological treatment option in VaD. These include suppressing proinflammatory cytokines such as IL-1β and TNF, increasing the expression of anti-inflammatory cytokines such as IL-10, suppressing glial activation and regulating inflammation-related signalling pathways such as the NF-κB, LCN2-JAK2/STAT3 and inflammasome pathways [62]. Such measures have shown a significant decline in cognitive impairment in rodent models of VaD. For instance, repeated administration of IL-1β in a CCH mouse model demonstrated protection against brain damage [63]. The central infusion of angiotensin IV, an important player in the regulation and control of blood pressure in a rat model of CCH showed a significant reduction in the expression of the pro-inflammatory cytokines IL-6, IL-12, IL-1β and TNF [64]. Therefore, the role of inflammation, both as a cause and effect has been illustrated and neuroinflammation inevitably plays a key role in contributing to the pathogenesis of VaD.

While the aforementioned pathological mechanisms drive major neuropathological hallmarks such as white matter lesions, neuronal cell death, and associated cognitive decline, these mechanisms in response to CCH may be significantly influenced by epigenetic modulation. The next section will explore the connection between CCH and epigenetic modulation in VCI and VaD.

## Epigenetics: Bridging the gap in mechanisms of CCH

Given the growing evidence of environmental factors and gene-environment interactions in the pathogenesis of VaD, epigenetics is emerging as a pivotal player in the disease. A deeper understanding of its role could provide valuable insights into VaD and pave the way for developing preventive treatments that target these gene-environment interactions through epigenetic mechanisms. Epigenetic changes involve heritable alterations of gene expression without any changes made to the deoxyribonucleic acid (DNA) sequence itself [65]. These are changes primarily due to environmental stimuli (e.g. stress, diet, smoking, alcohol consumption and work environment) and such epigenetic changes are reversible [66]. The three main mechanisms of epigenetics are namely DNA methylation, histone modifications and non-coding RNA synthesis [67]. These epigenetic mechanisms may function individually or have cross-talks amongst each other to eventually regulate transcriptional gene expression. The chromatin structure is modified by a process termed chromatin remodelling via several mechanisms that include enzyme-induced covalent modification and repositioning of nucleosomes to facilitate the regulation of gene transcription [68]. The chromatin is arranged from a condensed to a decondensed state, providing access for the transcriptional machinery to modulate gene expression. The basal transcriptional machinery primarily involves the RNA polymerase II and transcription factors IIA (TFIIA), TFIIB, TFIID, TFIIE, TFIIF and TFIIH [69]. While the epigenetic mechanisms do not change the basal transcriptional machinery, they alter the gene expression by recruiting proteins or by inhibiting the transcription factors from binding to form the transcription initiation complex [70]. Hence, by having dynamic control of the chromatin structure it allows for precise regulation of cellular processes such as gene expression [71].

Findings from several studies serve as evidence for epigenetic contributions to the pathophysiology of dementia in general. These include studies where DNA methylation and hydroxymethylation were observed to be significantly reduced in the hippocampus, entorhinal cortex, cerebellum, prefrontal cortex of AD patients compared to healthy controls [65]. An interesting study is that of monozygotic twins where one developed dementia while the other did not. Despite having the same genetic makeup, growth environment and educational qualifications, they displayed differences in DNA methylation levels. The individual with dementia had significant reduction in DNA methylation and hydroxymethylation in their brain cortical neurons [72]. It was identified that the twin who developed dementia was exposed to pesticides at workplace while the other did not, thus emphasising the effect of the environment on epigenetics which in turn contributed to the development of dementia. Recently, a large-scale epigenetic study was conducted involving six DNA methylomics studies of AD where they mapped the differentially methylated loci to different brain regions in accordance with Braak staging [73]. Out of which, 84 novel genes were identified to be differentially methylated in the cortex region.

With postulations of the role of epigenetics in driving the etiology of AD, several epigenome-wide association studies (EWAS) were conducted on AD brain samples over recent years. Meta analyses of six such independent EWAS studies, resulting in the identification of novel differentially methylated loci specific to AD [73]. Specifically, this study suggests that DNA methylomic changes in AD are specific to cortex cell types [73]. While no EWAS studies are yet available from VCI/VaD patients, there is evidence demonstrating that epigenetics, particularly DNA methylation, plays a key role in promoting vascular aging and cerebrovascular diseases [74]. Age-associated changes in vascular cells, along with conditions such as diabetes and hypertension, may promote alterations in the epigenetic landscape of brain microvessels. These epigenetic changes could lead to dysregulated gene expression and vascular dysfunction, potentially contributing to CCH. Such epigenetic contributions are pivotal in understanding and elucidating the pathogenesis of VCI and VaD. While the role of epigenetics in AD, Parkinson’s disease (PD), frontotemporal dementia (FTD) and Lewy body dementia (LBD) are increasingly studied and reviewed, the same cannot be said for VaD [3,65,75,76]. Moreover, the heterogeneity in the models used to study VaD and its representativeness of the symptoms in VaD patients further convolutes the landscape. It is therefore imperative to understand the role of the three different epigenetic mechanisms and their regulation in VCI and VaD. Recent studies by our group have provided insights into how CCH may drive changes in the DNA methylome and contribute to disease pathology in a mouse model of VaD [77]. In the next section, we will explore how epigenetic changes may play a pivotal role in the development and progression of VaD.

## Epigenetic Mechanisms in VaD

### Role of DNA Methylation in VaD

#### Understanding the components of DNA methylation

Amongst the three epigenetic mechanisms, DNA methylation is the most upstream of the hierarchy of occurrence in the central dogma. It is also the most widely studied epigenetic change which regulates cellular processes [78]. DNA methylation involves the covalent transfer of a methyl group onto the C5 position of the cytosine ring of DNA [70]. DNA methylation is generally associated with transcriptional repression of gene expression. This direct chemical modification to DNA is executed by a family of enzymes known as DNA methyltransferases (DNMTs). Specifically, DNMTs catalyse the DNA methylation process by transferring a methyl group from S-adenyl methionine (SAM) to the fifth carbon of a cytosine residue forming 5-methylcytosine (5mC). In particular, DNMT1 is broadly involved in methylation maintenance where it preferentially methylates hemi-methylated sites during DNA replication on newly replicated strands [79]. In contrast to other DNMTs, DNMT2 is involved in the methylation of small RNA molecules and its function is yet to be well characterised [80]. DNMTs 3A and 3B are involved in *de novo* methylation where it methylates unmodified DNA, forming new DNA methylation patterns [79]. DNMT3-Like (DNMT3L), which is specifically expressed in oocytes, also assists DNMT3A and DNMT3B to increase their ability to bind to the methyl group donor SAM though it does not have any catalytic activity [81]. Currently, amongst all DNMTs, it is mainly DNMT1, DNMT3A and DNMT3B that are crucially involved in genomic integrity as they are known to be required for transcriptional silencing [70]. These three DNMTs are heavily involved in embryonic development where their expression is reduced upon terminal differentiation [81]. However, the DNA methylation patterns in post-mitotic neurons are not stable. In fact, post-mitotic neurons in mature mammalian brains have substantial expression of DNMTs, hinting that they could play a crucial role in the brain [70]. Additionally, evidence shows that alterations in gene expression followed by neuronal depolarisation also undergo alterations in DNA methylation patterns [82].

#### The dynamic mechanism of DNA methylation

Given the reversible nature of epigenetic changes, DNA methylation changes are also subject to these reversible ‘tags’ in the form of DNA demethylation. DNA demethylation, which involves the conversion of 5mC to its unmodified state, can be classified as either passive or active. Passive DNA demethylation occurs in dividing cells when DNMTs are absent or inactive to methylate DNA following each cell cycle of division, thus diluting the global content of 5mC over several rounds of replication [83]. On the other hand, active DNA demethylation occurs in both dividing and non-dividing cells where it involves catalytic activity to remove methylated cytosine [83]. With the covalent bonds between the carbon groups being too strong to be cleaved directly from 5mC to C, DNA demethylation occurs through a complex series of chemical reactions [70]. Several mechanisms have been proposed that facilitate active DNA demethylation, which includes enzymatic removal of methyl groups from 5mC (via methyl-CpG-binding domain protein 2), base excision repair (BER) through direct excision of 5mC, deamination of 5mC to thymine followed by BER, nucleotide excision repair, radical SAM mechanism and oxidative demethylation [84]. In recent years, the ten eleven translocation (TET)-mediated oxidative demethylation in combination with thymine DNA glycosylase (TDG)-dependent BER has been reported to play a key role in mediating active DNA demethylation. The family of TET enzymes (TETs 1-3) function through oxidation of 5mC to 5-hydroxymethylcytosine (5hmC), 5-formylcytosine (5fC) or 5-carboxylcytosine (5caC), which then act as a substrate for TDG-dependent BER to replace 5mC with C [85]. TET proteins are multidomain enzymes containing a core catalytic region that preferentially binds to CG nucleotide (CpG)-rich islands, but does not interact with the surrounding DNA bases [83]. TET1 has been specifically observed to be enriched in CpG-rich regions, active and bivalent promoters [86]. In addition to this mechanism, it is notable that DNMT1’s interactive partners UHRF1 and UHRF2 have been observed to bind 5hmC DNA, suggesting that DNA methylation is maintained by perhaps recruiting DNMT1 to hemi-hydroxymethylated sites [83]. The role of 5hmC is therefore complex but interesting. TET proteins, specifically TET1 and has been implicated in neuronal functions, and a high abundance of 5hmC levels have been observed in various subtypes of neurons [86].

#### Evidence for DNA methylation in VaD

In the context of VaD, only five studies have thus far explored the DNA methylation landscape in animal models (Table 1). While genome-wide DNA methylation data is not available in VaD patients, global DNA methylation patterns are available from the plasma of VaD patients. Not only was there a trend of higher DNA methylation observed in VaD patients, but they also had a significantly higher concentration of homocysteine and methylmalonic acid, and a lower folate and plasma 5-methyltetrahydrofolate, which were associated with global DNA methylation levels [87]. A specific DNA methylation analysis was also performed in post-mortem brain tissues of VaD patients alongside other dementia patients (AD, DLB, Huntington’s disease (HD), PD) to help quantify the DNA methylation level of Ank1, which was significantly hypermethylated in the epigenome-wide studies of AD. Ank1 hypermethylation was observed in the entorhinal cortex of VaD patients, but only with co-existing AD pathology [88]. Despite being a useful indication of pathology in VaD, Ank1 does not hold promise as a biomarker due to the lack of specificity, and was not detected in pre-mortem blood samples even in AD patients [89]. In relation to animal studies, there were only two studies on global DNA methylation levels, and one on genome-wide DNA methylation (Table 1). Both global DNA methylation studies were conducted by the same group where they reported consistent results of reduced global methylation levels 10 days post inducing CCH while this trend was reversed at the 90-day timepoint [90,91]. However, DNMT3A levels with respect to the sham controls was inconsistent in both studies with one showing an increase and the other a decrease at the 90-day timepoint. Intravenous administration of SAM in a rat model of CCH was shown to improve spatial memory, although the global DNA methylation levels remained unaltered [91]. A genome-wide DNA methylation analysis in a rat model of VaD showed a relative hypomethylation level in the promoter gene region and identified 1180 differentially methylated genes where the cluster of interacting genes were strongly associated with the vascular endothelial growth factor (VEGF) pathway [78].

**Table 1:** Role of DNA Methylation in Vascular Dementia.

	Article	Sample	Experimental info.	Findings	Ref.
**1.**	Chronic cerebrovascular hypoperfusion affects global DNA methylation and histone acetylation in rat brain (Repeated in histone modifications section)	2-VO surgery, Sprague Dawley rats aged 23 weeks	Changes of global DNA methylation and histone acetylation levels	10 days post 2-VO surgery •↓ Global methylation levels •↓ Histone H3 90 days post 2-VO surgery •↑ Global methylation levels •↓ DNMT3A •↑ MBD2 •↓ Histone H3 •↓ HDAC3 •↑ cAMP	[51]
**2.**	Epigenetic signature of chronic cerebral hypoperfusion and beneficial effects of S-adenosylmethionine in rats (Repeated in histone modifications section)	2-VO surgery, Sprague Dawley rats aged 23 weeks (*in vivo)*	DNA methylation, histone acetylation, SAM cycle were monitored	10 days post 2-VO surgery •↓ Global methylation levels 90 and 180 days post 2-VO surgery •↑ Global methylation levels •↑ DNMT3A •Altered SAM cycle •↑ Histone global H4 acetylation •↑p300/CREB-binding protein (CBP) → Histone acetyltransferase •↑BDNF •↓HDACs	[77]
**3.**	Genome-wide DNA methylation profiling in a rat model with vascular dementia	BCCAO surgery, Wistar rats	To identify candidate genes that undergo changes in hippocampal DNA methylation under VaD	•1180 differentially methylated genes •Relative hypomethylation in the promoter region of the VaD model •↑ mRNA expression of hippocampal genes vascular endothelial growth factor (VEGFA) and kinase insert domain receptor	[78]
**4.**	A cross-brain regions study of ANK1 DNA methylation in different neurodegenerative diseases	Post-mortem brain tissue (AD, DLB, VaD, HD, PD and non-demeneted control subjects)	To quantify DNA methylation levels across a 118bp region of ANK1 in different neurodegenerative diseases	•ANK1 hypermethylation in entorhinal cortex in AD, HD and PD •Elevated ANK1 DNA methylation (i.e. hypermethylation) in VaD and DLB only with co-existing AD pathology	[87]
**5.**	Homocysteine metabolism and the associations of global DNA methylation with selected gene polymorphisms and nutritional factors in patients with dementia	Human plasma samples (From AD, VaD and mixed dementia patients)	To investigate the association of global DNA methylation, homocysteine, folate and vitamin B12 status with dementia	•Dementia patients had significantly higher concentrations of homocysteine, methylmalonic acid and lower folate and plasma 5-methyltetrahydrofolate •No difference in DNA methylation between patients and controls •A trend of higher DNA methylation in VaD patients observed •Global DNA methylation was associated with markers of folate status, creatinine, glucose and PON1 and ILB1 polymorphisms	[88]

While these data provide a basis for understanding the global patterns of DNA methylation in VaD, they lack the rigor to provide a mechanistic understanding of VaD pathogenesis, and the key gene clusters involved in driving VaD progression. Moreover, all three above mentioned animal studies utilized an occlusion model (2-Vessel Occlusion (2-VO) or Bilateral Common Carotid Artery Occlusion (BCCAO) surgery), where there was a complete blockage of blood vessels inducing thus incurring acute damage [51,78]. This does not represent the CCH state observed in human patients and results in damage to the visual pathway, unlike that in humans. Nevertheless, a recently published study by our group investigated the role of DNA methylation in the CCH mouse model. We have reported the modulation of DNA methylation landscape both globally and with respect to differentially methylated genes implying its role in the pathogenesis of VaD [77].

**Figure 2.**
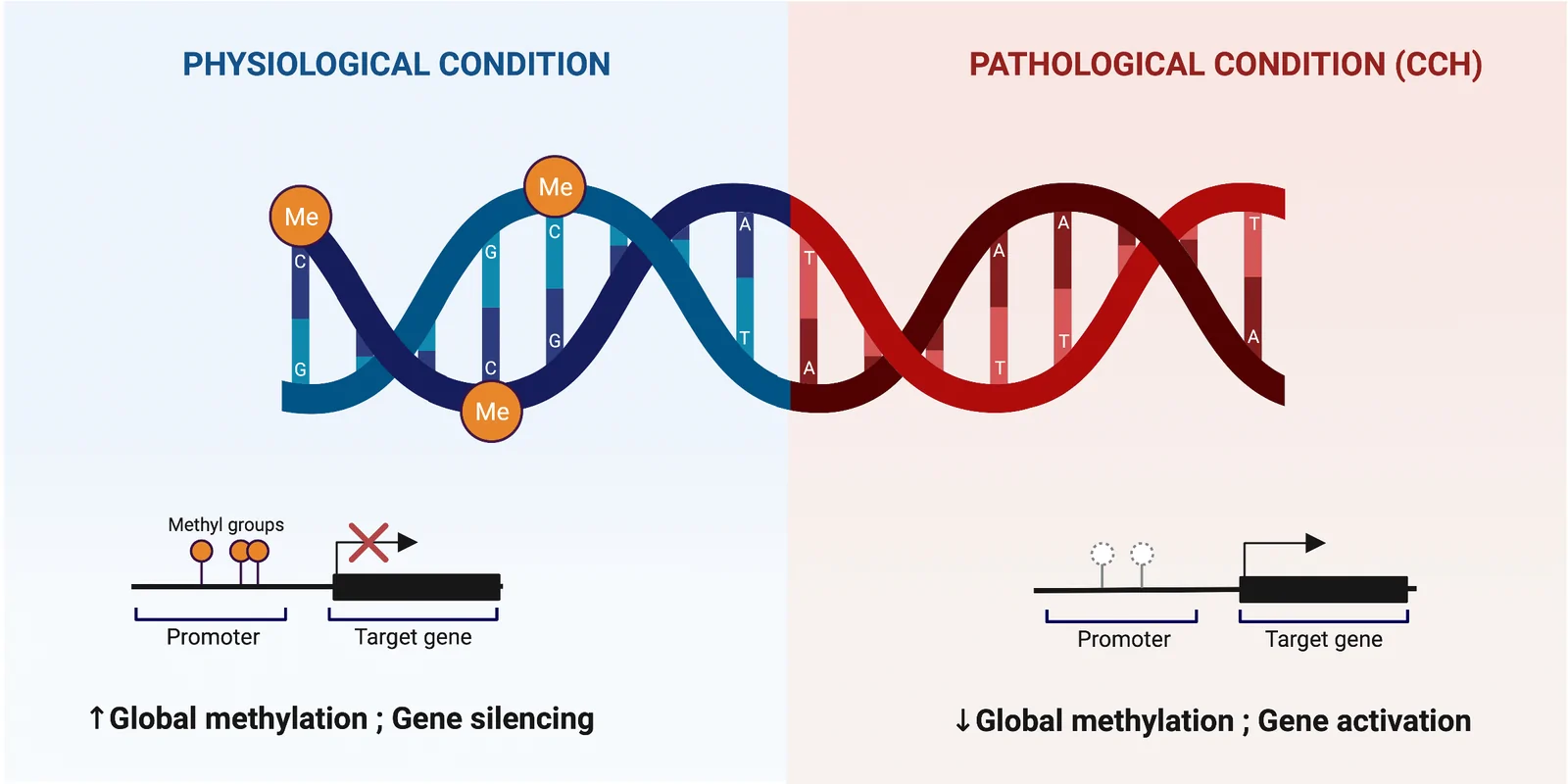
**Illustration of the role of DNA methylation in the pathophysiology of VaD**. The global methylation status under physiological condition and pathological conditions have been shown. A respective hypermethylated (increase in addition of methyl groups) and hypomethylated (decrease in addition of methyl groups) status have been demonstrated with the implications on the regulation of gene expression. This is based on the limited existing literature in the nascent field of DNA methylation under CCH. The methylation status may vary depending on the respective genes and gene context.

The role of DNA methylation in VaD is thus promising but the field is rather nascent (Fig. 2). Apart from global methylation trends measuring the levels of DNMTs, TETs and 5hmC, the difference in brain-regions and cell-types could also be analysed. In terms of DNA methylation sequencing, a whole genome bisulfite sequencing of post-mortem VaD patient brains and a representative mouse model of VaD, the bilateral common carotid artery stenosis (BCAS), could be performed. Future global methylation analysis should be focused on identifying differentially methylated gene regions and subsequent gene clusters pertinent to VaD. There is thus scope for extensive work to unravel the role of DNA methylation in the pathogenesis of VaD.

### Role of Histone Modifications in VaD

#### Histones and their role in regulating transcription

Histones are highly conserved, positively charged proteins that bind strongly to the negatively charged DNA and compresses it tightly within the nucleus to form chromatin which then facilitates regulation of gene transcription [92]. The basic unit of chromatin is the nucleosome which is comprised of approximately 147 base pairs of DNA being wrapped around an octamer of two of each core histones H2A, H2B, H3 and H4 whereby this unit is repeated throughout the genome with the H1 linker protein stabilising internucleosomal DNA [93]. The interaction between the DNA and histones are mediated by the amino-(N)-terminal tail of histone proteins where a specific pattern of modifications occurs. Histones are post-translationally modified and such modifications are pivotal in controlling chromatin structure and regulation of gene transcription [94]. For instance, modifications which disrupt the histone-DNA interaction cause the nucleosomes to unwind, resulting in an open chromatin formation known as euchromatin. The transcriptional machinery thus binds easily to the DNA which is accessible, allowing gene expression to occur. On the contrary, modifications that strengthen the histone-DNA interaction causes the nucleosomes to pack tighter, resulting in a closed chromatin formation known as heterochromatin. This structure makes the DNA inaccessible to the transcriptional machinery, thus resulting in gene silencing. The post-translational modifications of histones are generally classified into acetylation, methylation, phosphorylation and ubiquitination [93]. In fact, histone acetylation and histone methylation are the most well characterised epigenetic modifications after DNA methylation [95].

### Acetylation

Histone acetylation and deacetylation refer to the transfer and removal of an acetyl group to the lysine groups on the N-terminal tails of histones by histone acetyltransferases (HATs) and histone deacetyltransferases (HDACs), respectively [96]. Acetylation of lysine neutralises the charge on histones, therefore reducing the strength of interaction with DNA which is negatively charged resulting in a euchromatin state. Conversely, deacetylation of histones gives the opposite effect.

### Methylation

Histone methylation and demethylation refers to the addition and removal of methyl groups on the basic residues of histone such as arginines, lysines and histidines by histone methyltransferases and demethylases, respectively [97]. Unlike histone acetylation, histone methylation does not alter the charge of histones but rather determines the active and silent states of surrounding genes via modulation of the chromatin architecture. Histone methylation also takes different forms where lysines can be mono-, di- or tri-methylated whereas arginines can be mono-methylated, symmetrically or asymmetrically dimethylated and histidines can be monomethylated [97]. These different forms of histone methylation are context-dependent and hence functionally diverse. Apart from the degree and location of methylation, the chromatin effector molecules (i.e. ‘readers’ of the histone modification) could also play a part in determining the function of methylation [97]. In fact, in some cases the same modification can be associated with contrasting activities of transcriptional activation and repression.

### Phosphorylation

Histone phosphorylation and dephosphorylation involves the addition and removal of phosphate groups to the N-terminal of histone tails on residues such as serine, threonine and tyrosine mediated by protein kinases and phosphatases, respectively [98]. Similar to histone acetylation, histone phosphorylation is generally associated with transcriptional activation. This is due to the phosphate groups being negatively charged, thus repelling DNA interaction, resulting in a euchromatin state, allowing access to the transcriptional machinery. Histone phosphorylation is also observed to promote acetylation by recruiting HATs [98]. Crosstalk within and between histone modifications have been demonstrated which culminate to the important roles it plays such as in chromatin condensation, transcriptional regulation and DNA repair [99].

### Ubiquitylation

Histone ubiquitylation and deubiquitylation refers to the addition and removal of a ubiquitin moiety to the lysine residues of histone core proteins by the sequential action of E1-activating, E2-conjugating and E3-ligating enzymes and isopeptidases, respectively [100]. The enzymes determine substrate specificity and whether the lysine residue is mono- or poly-ubiquitylated where the most common types are mono-ubiquitylated H2A and H2B [101]. It is postulated that the addition of ubiquitin which is a 76-amino acid protein, provides significant steric bulk to the nucleosome, thus increasing the surface area of exposure for the binding of the translational machinery to regulate transcription [102]. Apart from transcriptional regulation, an important function of histone ubiquitylation is to maintain genome stability by regulating the DNA repair response [103]. Crosstalk between histone ubiquitylation and other histone modifications have been observed and, in some cases, the addition of ubiquitin acts as a signal for proteolysis of methylated histones [104].

#### Evidence for Histone modifications in VaD

**Figure 3.**
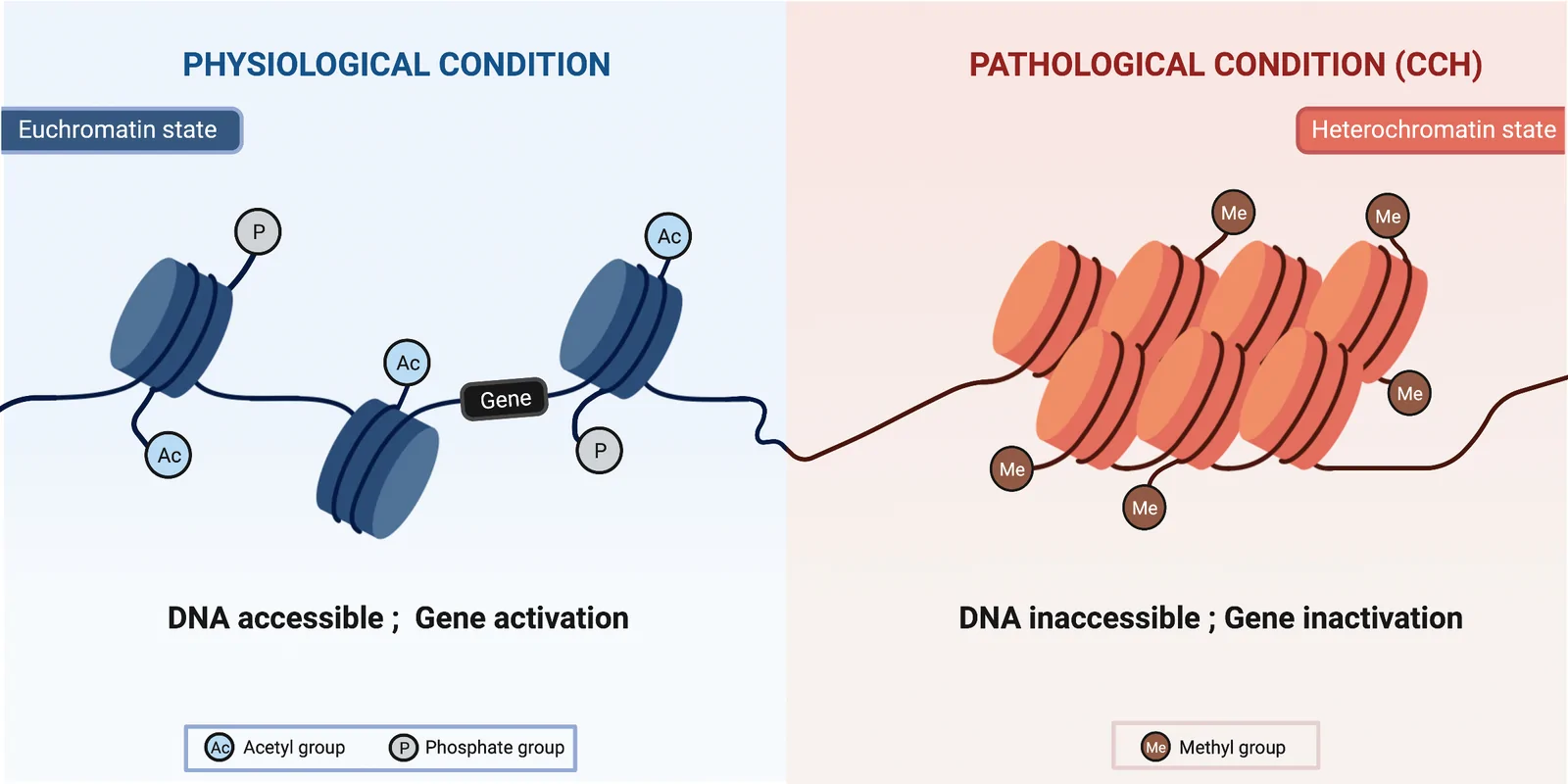
**Illustration of the role of histone modifications in the pathophysiology of VaD**. Based on the existing evidence in literature, the chromatin under physiological conditions is known to be in the lightly packed conformation known as the euchromatin state with the presence of negatively charged histone modifications comprising of groups such as acetyl and phosphate groups. Under pathological conditions such as that under CCH, the chromatin is in a tightly packed conformation known as the heterochromatin state with the presence of methyl groups. The DNA is therefore more accessible by the transcriptional machinery in the euchromatin state compared to that in the heterochromatin state, activating the gene.

Histone modifications have been demonstrated to occur in neurodegenerative diseases and are thus being increasingly studied to deduce possible pharmacological targets as treatment options [105]. Histone modifications also play a role in atherosclerosis, which increases the risk of VaD by resulting in a cerebral hypoperfused state. In fact, the HDAC variant HDAC9 (allele 7p21.1) was shown to be associated with promoting atherosclerosis possibly by promoting plaque development and raising the risk of subsequent occurrence of thromboembolism [106]. The deficiency of the same variant was shown to attenuate atherosclerosis [107]. In terms of CCH in VaD, approximately 12 studies are exploring the role of histone modifications in animal models of VaD and 1 study on VaD patients (Table 2). Pertaining to histone acetylation levels, a study by Wu and colleagues reported an increase in HATs, global H3 and H4 acetylation levels and a decrease in HDAC3 under CCH [91]. However, an earlier study by the same group had reported a decrease in H3 acetylation levels and no significant difference in H4 acetylation levels under CCH [90]. Similarly, another study which was conducted in an ischemic *in vitro* model, showed hypoacetylation of H3 and H4 levels [108]. The latter studies are consistent with the theoretical understanding in how histone acetylation could potentially play a role in VaD. The only available human study thus far on the role of histones in VaD shows that high titres of antibodies against histones are detected in the blood samples of VaD patients [109]. This was postulated to be reflective of the disruption of membrane fluidity and integrity, compromised blood-brain barrier and aberrant immune functions in dementia patients [109]. Such global analyses of histone levels and their modifications furbish the understanding that increased expression of HDACs under CCH reduces histone acetylation, resulting in a heterochromatin state that inhibits gene transcription (Fig. 3).

**Table 2:** Role of Histone Modifications in Vascular Dementia.

	Article	Sample	Experimental info.	Findings	Ref.
**1.**	Epigenetic signature of chronic cerebral hypoperfusion and beneficial effects of S-adenosylmethionine in rats	2-VO surgery, Sprague Dawley rats aged 23 weeks (*in vivo)*	DNA methylation, histone acetylation, SAM cycle were monitored	•↑ DNMT3A •Altered SAM cycle •↑ Histone global H4 acetylation •↑p300/CREB-binding protein (CBP) → Histone acetyltransferase •↑BDNF •↓HDACs	[90]
**2.**	Epigenetic Features Induced by Ischemia-Hypoxia in Cultured Rat Astrocytes	Cultured astrocytes (*in vitro)*	DNA methylation, histone acetylation, SAM cycle were monitored in astrocytes	•↑ Global hypermethylation •↑ DNMT1, DNMT3A •Hypoacetylation of histones H3/H4	[91]
**3.**	HDAC inhibitor protects chronic cerebral hypoperfusion and oxygen-glucose deprivation injuries via H3K14 and H4K5 acetylation-mediated BDNF expression	Modified BCCAO surgery (*in vivo)*; SH-SY5Y neuroblastoma cells (OGD, *in vitro)*	Testing of HDAC inhibitors against in vivo and in vitro models of CCH	•Compound 13 found to be effective •↑ BDNF levels •↑ Acetylation of H3K14 and H4K5 •↑ of BDNF specific isoform expressions to reduce ischemic and hypoxic injuries.	[108]
**4.**	Chronic cerebrovascular hypoperfusion affects global DNA methylation and histone acetylation in rat brain	2-VO surgery, Sprague Dawley rats aged 23 weeks	Changes of global DNA methylation and histone acetylation levels	10 days post 2-VO surgery •↓ Global methylation levels •↓ Histone H3 acetylation 90 days post 2-VO surgery •↑ Global methylation levels •↓ DNMT3A •↑ MBD2 •↓ Histone H3 acetylation •↓ HDAC3 •↑ cAMP	[109]
**5.**	Ginsenoside Reduces Cognitive Impairment During Chronic Cerebral Hypoperfusion Through Brain-Derived Neurotrophic Factor Regulated by Epigenetic Modulation	BCAS surgery, C57BL/6J mice aged 5-7 weeks	Elucidating neuroprotective effects of Ginsenoside Rd (GSRd - one of the main active ingredient in Panax ginseng) in the context of CCH	Under CCH only, •↑ Impairment of learning and memory behaviours (Morris Water Maze) •↓ Neuron survival •↓ BDNF expression •↓p300/CBP •↑HDAC2	[110]
**6.**	Post-occlusion administration of sodium butyrate attenuates cognitive impairment in a rat model of chronic cerebral hypoperfusion	2-VO surgery, Sprague Dawley rats	Investigating the effect of sodium butyrate (SB- HDAC inhibitor) in CCH	Under SB, •↓ Hippocampal-dependent spatial learning disability (Novel Object Recognition Test) •↑ HDAC1/2 mRNA level •↑ Histone H4 acetylation •↑ Nrf2 transcriptional activation in hippocampus	[111]
**7.**	Cognitive Improvement Induced by Environment Enrichment in Chronic Cerebral Hypoperfusion Rats: a Result of Upregulated Endogenous Neuroprotection?	2-VO surgery, Wistar rats aged 7-weeks old	Investigating effect of environmental enrichment on cognitive function, brain histone acetylation levels, neuroprotection-related transcription factors, oxidative stress and histological changes in the brain	Under CCH only •↑ Oxidative damage •↑ Histopathological damage •↑ Cognitive impairment (Morris Water Maze) •↑ HIF1α •↓ p-CREB •↑ GFAP in corpus callosum, CA1, CA3 and dentate gyrus •↑ IBA1 in cortex, CA1, CA3, dentate gyrus	[112]
**8.**	Protective effects of 10,11-dihydro-5H-dibenzo[b,f]azepine hydroxamates on vascular cognitive impairment	Modified BCCAO surgery, C57BL/6J mice aged 16 weeks	Investigating the effect of 10,11-dihydro-5H-dibenzo[b,f]azepine hydroxamates (HDAC inhibitors) on VCI	Under HDAC inhibitor, •↓ Cognitive impairment •Improved hippocampal atrophy •↑ Histone acetylation of H3K14 or H4K5 in cortex and hippocampus	[113]
**9.**	HDACi protects against vascular cognitive impairment from CCH injury via induction of BDNF-related AMPA receptor activation	BCCAO surgery, C57BL/6J mice aged 16 weeks	Investigating whether BDNF activation by HDAC inhibitor may affect protein levels of AMPA and dopamine receptors under CCH conditions	Under CCH and introduction of HDAC inhibitor, •↑ BDNF •↑ AMPARs •↑ Protection against hippocampal atrophy and cognitive impairment •Restoration of CBF	[114]
**10.**	Attenuation of vascular dementia by sodium butyrate in streptozotocin diabetic rats	Streptozotocin (STZ) injected Albino Wistar rats	Investigating the effect of sodium butyrate (HDAC inhibitor) on STZ diabetes induced vascular dementia	Under SB, •↓ Diabetes induced impairment of learning, memory •↓ Impairment of endothelial function	[115]
**11.**	Donepezil attenuates vascular dementia in rats through increasing BDNF induced by reducing HDAC6 nuclear translocation	BCCAO surgery, Sprague Dawley rats	To find out the mechanism by which Donepezil attenuates vascular dementia disease progression	Under Donepezil, •↑ BDNF in cortex and hippocampus •↓ Nuclear translocation of HDAC6 •↓ Binding between HDAC6 and BDNF promoter IV in cortex	[116]
**12.**	Arsenic toxicity induced endothelial dysfunction and dementia: pharmacological interdiction by histone deacetylase and inducible nitric oxide synthase inhibitors	Arsenic administered through drinking water, Albino Wistar rats	Investigating the potential of sodium butyrate (HDAC inhibitor) and aminoguanidine (iNOS inhibitor) in preventing arsenic toxicity induced vascular endothelial dysfunction and dementia	Under SB and Aminoguanidine, •↓ Impairment in learning and memory •↓ Endothelial function	[117]
**13.**	Antihistone and anti-dsDNA autoantibodies in Alzheimer's disease and vascular dementia	Human patients	To evaluate the link between the presence of histones, dsDNA and senile dementia	•High titers of antibodies against histones	[118]

HDAC inhibitors (HDACi) have therefore been increasingly explored as a possible intervention. In fact, HDACi have been established to assert neuroprotective effects and improve cognition in animal models of dementia [110]. The neuroprotective effects of HDACi have been echoed in CCH as well where studies have shown that administration of sodium butyrate (SB), a HDACi, has been shown to significantly reduce memory impairment and endothelial dysfunction in rat models [110,111,112]. Apart from SB, there are other HDACi whose efficacy in combating CCH are being explored. Kaur and colleagues identified the protective effects of 10,11-dihydro-5H-dibenzo[b,f]azepine hydroxymates, specifically compound 13, in attenuating cognitive impairment and hippocampal atrophy, and increasing histone acetylation of H3K14 and H4K5 [113]. A follow-up study by the same group investigating compound 13 in both *in vivo* and *in vitro* models showed that the protection conferred by the HDACi in CCH is mediated through H3K14 and H4K5 acetylation by increasing expression of specific brain-derived neurotropic isoforms [114]. They further probed into the downstream molecular mechanism to elucidate that compound 13 protects against CCH by BDNF-related AMPA receptor activation where apart from increased protection against cognitive impairment and hippocampal atrophy, cerebral blood flow was also restored [115]. Similarly, HDACi like ginsenoside Rd (the main active component of ginsenosides), donepezil (cholinesterase inhibitor) and even environmental enrichment have shown to attenuate cognitive impairment and increase neuroprotection [116,117,118].

The role of histone modifications in the context of VaD is therefore of increasing interest and scope to explore this mechanism further. Apart from global histone, HAT and HDAC levels, efforts to attenuate cognitive impairment in CCH through various HDACi is evident. However, its effectiveness has to be further validated and the molecular mechanism underlying such interventions remain elusive. Nevertheless, the emphasis on ensuring an appropriate balance between stable and dynamic histone modifications to confer protection against VaD is clear.

### Role of non-coding RNAs (ncRNAs) in VaD

#### Understanding the classifications of ncRNAs and their role in neurodegenerative diseases

**Figure 4.**
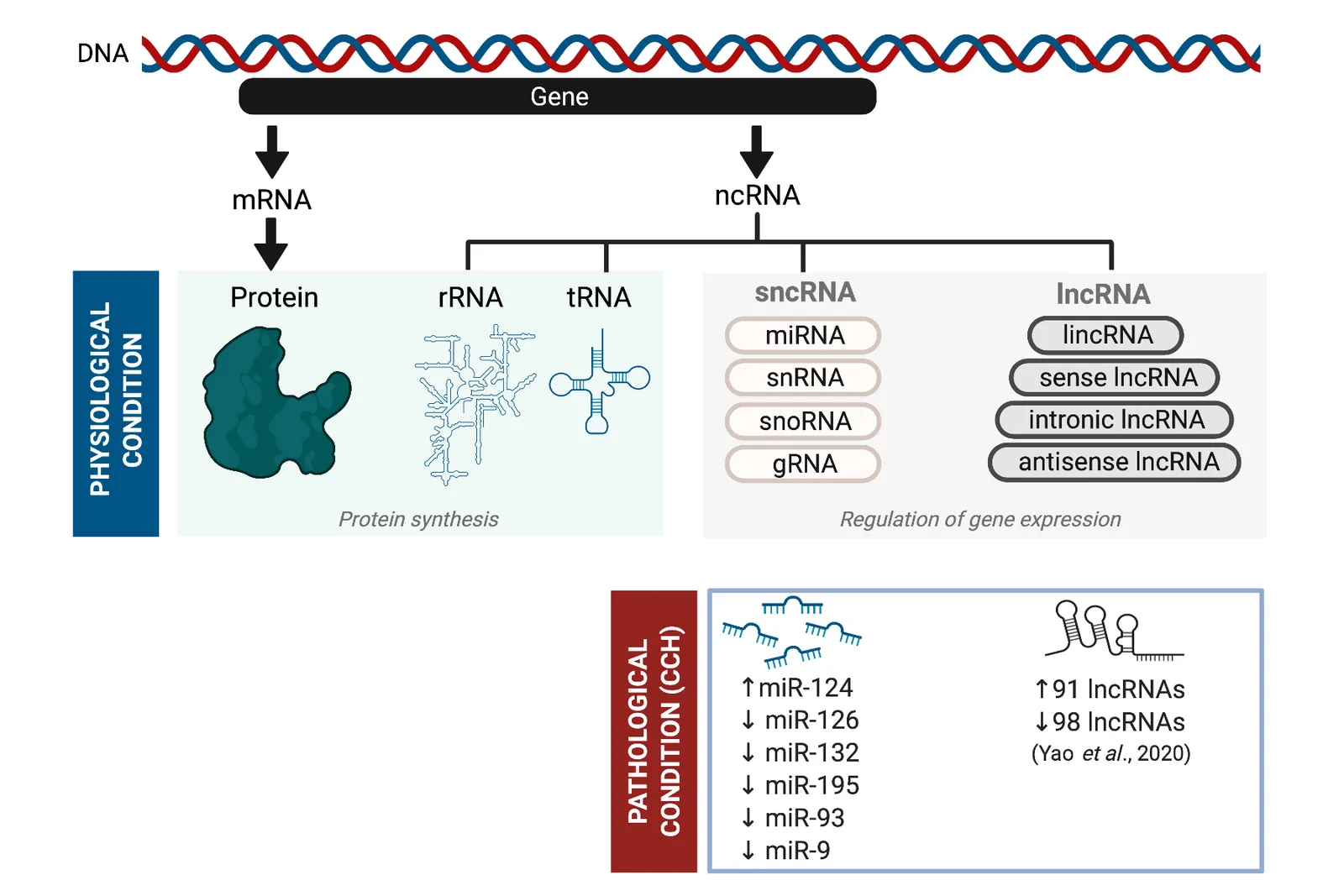
**Illustration of the role of non-coding RNAs (ncRNAs) in the pathophysiology of VaD**. RNAs translated to proteins are known as messenger RNAs (mRNAs) while those which are not translated to proteins are known as ncRNAs. Based on existing evidence in the literature, the classifications of ncRNAs are represented. ncRNAs comprise of ribosomal RNA (rRNA), transfer RNA (tRNA) which are both involved in protein synthesis and small ncRNAs (sncRNAs) and long ncRNAs (lncRNAs) which are both involved in regulation of gene expression though previously labelled to be “junk”. sncRNAs further include microRNAs (miRNAs), small nuclear RNAs (snRNAs), small nucleolar RNAs (snoRNAs), guide RNAs (gRNAs) while lncRNAs include intergenic lncRNAs (lincRNAs), intron lncRNA (intronic lncRNA), and sense and antisense lncRNA (sense lncRNA; antisense lncRNA). Some of the reported differential miRNA levels and lncRNAs under CCH are also included in the diagram.

Non-coding ribonucleic acids (ncRNAs) refer to a class of RNAs that are not translated to form a functional protein [119]. Accounting for approximately 98-99% of the transcripts in the human genome, ncRNAs were considered to be “junk” due to their non-coding nature till their biological functions and relevance were brought to light [120]. The field of ncRNAs is rather nascent but the research is expanding and the functions of ncRNAs are increasingly uncovered. To date, the functions of ncRNAs are wide-ranging from being involved in gene regulation, transposon control, translational repression and chromatin modifications [121]. ncRNAs are generally classified as ribosomal RNA (rRNA), transfer RNA (tRNA), small ncRNAs (sncRNAs) which includes microRNAs (miRNAs), small nuclear RNAs (snRNAs), small nucleolar RNAs (snoRNAs), guide RNAs (gRNAs) and long ncRNAs (lncRNAs) that include intergenic lncRNAs (lincRNAs), intron lncRNA (intronic lncRNA), and sense and antisense lncRNA (sense lncRNA; antisense lncRNA) [122]. In general, sncRNAs are less than 200 base pairs while lncRNAs are more than 200 base pairs, respectively (Fig. 4). Out of these ncRNAs, miRNAs are the most studied sncRNAs, which hold potential as biomarkers of neurodegenerative diseases and exhibit specific signalling in the brain [120]. Apart from the close association between miRNAs and neurodegenerative diseases, the mechanism by which it plays a role in biological functions is heavily investigated upon. In fact, miRNAs have been implicated to play a role in transcriptional gene regulation by means of RNA silencing and RNA interference. Transcriptional gene silencing by miRNAs have been found to be mediated through small-RNA silencing pathways where promoter-directed miRNAs mediate repressive chromatin modifications [123]. The chromatin states are postulated to be inherited during chromosome duplication. While the exact mechanism remains elusive, retention of old histones during DNA replication and RNA silencing induced a heterochromatin state which has the property of being inherited through cell divisions that serve as evidence of epigenetic inheritance [123]. Apart from miRNAs, mutation and abnormal regulation of lncRNAs have also been shown to play a key role in neurodegenerative diseases [124]. While the number of functional lncRNAs are still debated, some of the functions include regulating chromatin architecture and gene transcription, responding to DNA damage and repair, and in DNA replication [125].

#### Evidence for ncRNAs in VaD

ncRNAs, miRNAs and lncRNAs in particular, have been increasingly shown to play an emerging role in dementia such as VaD [126]. At present, ongoing research endeavors are exploring the role of ncRNAs in VaD across a total of 28 studies (Table 3). miRNAs are known to be ideal mediators of hypoxic stress responses that facilitates cellular adaptation to long-term hypoxia [127]. Zhang and colleagues summarised the roles of 14 regulatory miRNAs identified in the context of vascular cognitive impairment and dementia which were wide-ranging from conferring protection against CCH-induced cognitive impairment to regulating both synapse numbers and plasticity [128]. miRNAs play a key role in regulating complex cellular biological networks [129]. For instance, miR-124 has been shown to be significantly increased in ischemic conditions that confer protection of neurons against apoptosis. Similarly, its inhibition has been demonstrated to upregulate β-site amyloid precursor protein cleaving enzyme 1 (BACE1) in the hippocampus under CCH both in dementia patients and rat models, implying a regulatory role of miR-124 [127]. miR-126, on the other hand, was observed to mediate cognitive impairment in a mouse model of CCH when its endothelial expression was reduced [129]. miR-9, like miR-126, exhibited its regulatory role of BACE1 under CCH [130]. Many other miRNAs have been implicated in the pathogenesis of VaD. Several studies have highlighted that a reduction in miR-195 was observed under CCH and that increasing miR-195 levels protected against dementia that was induced by CCH [131,132,133,134]. Some of the mechanistic explanations for this phenomenon included preventing dendritic degeneration and neuronal death [135].

**Table 3:** Role of Non-coding RNA in Vascular Dementia.

	Article	Sample	Experimental aim	Findings	Ref.
**1.**	MicroRNA-195 prevents hippocampal microglial/macrophage polarization towards the M1 phenotype induced by chronic brain hypoperfusion through regulating CX3CL1/CX3CR1 signaling	2-VO surgery, Sprague Dawley rats	To investigate effect of cerebral circulation insufficiency on microglial polarisation after chronic brain hypoperfusion (CBH)	•↓ miR-195 in hippocampus involved in CBH-induced microglial polarisation towards M1 phenotype through regulation of CX3CL1 and CX3CR1 signalling	[127]
**2.**	Identification of a hippocampal lncRNA-regulating network in cognitive dysfunction caused by chronic cerebral hypoperfusion	2-VO surgery, Sprague Dawley rats	To elucidate mechanism of cognitive dysfunction caused by CCH.	•↑ 128 mRNAs, 91lncRNAs •↓ 108 mRNAs, 98 lncRNAs •Constructed competitive endogenous RNA network (559 lncRNA-miRNA-mRNA targeted pairs)	[128]
**3.**	MicroRNA-181c Ameliorates Cognitive Impairment Induced by Chronic Cerebral Hypoperfusion in Rats	2-VO surgery, Sprague Dawley rats	To find out the role of miR181c-TRIM2 pathway in learning and memory deficits induced by CCH	•↓ miR-181c continuously alongside ↑ TRIM2 in hippocampus of 2-VO model •Viral vector mediated miR181c delivery ameliorated cognitive impairment in CCH	[129]
**4.**	miR-124 Regulates the Expression of BACE1 in the Hippocampus Under Chronic Cerebral Hypoperfusion	2-VO surgery, Sprague Dawley rats	To find out miR-124 expression in the brain under CCH	•↓ miR-124, ↑BACE1 in the hippocampus of 2-VO model •Activation of EPAC-Rap1 pathway in inhibition of miR-124 under hypoxia or Aβ insult	[130]
**5.**	MicroRNA-153 impairs hippocampal synaptic vesicle trafficking via downregulation of synapsin I in rats following chronic cerebral hypoperfusion	2-VO surgery, Sprague Dawley rats	To investigate whether and how synaptic vesicle trafficking is impaired by CCH	•CCH impairs hippocampal glutamatergic vesicle trafficking by ↑miR-153 which in turn ↓ synapsin I at post-transcriptional level	[131]
**6.**	Nimodipine attenuates tau phosphorylation at Ser396 via miR-132/GSK-3β pathway in chronic cerebral hypoperfusion rats	2-VO surgery, Wistar rats	To investigate the potential mechanism of nimodipine in tauopathies induced CCH	•↓ miR-132 •↑ tau phosphorylation at Ser396 •Nimodipine attenuates CCH-induced tau phosphorylation by ↑miR-132 (inhibited activation of GSK-3β and neuronal apoptosis)	[132]
**7.**	MicroRNA-132 regulates total protein of Nav1.1 and Nav1.2 in the hippocampus and cortex of rat with chronic cerebral hypoperfusion	2-VO surgery, Sprague Dawley rats	To investigate the role of miR-132 in CCH	•↓ miR-132 in both hippocampus and cortex under CCH •Lentiviral mediated overexpression of miR-132 ameliorated dementia vulnerability induced by 2-VO •Over-expression of miR-132 inhibited Nav1.1 and Nav1.2	[133]
**8.**	miR-132 Down-regulates Methyl CpG Binding Protein 2 (MeCP2) During Cognitive Dysfunction Following Chronic Cerebral Hypoperfusion	2-VO surgery, Sprague Dawley rats	To investigate the status of MeCP2 expression after CCH and whether MeCP2 changes are associated with cognitive deficits after CCH	•↑ miR-132, ↓MeCP2 in hippocampus of 2-VO model	[134]
**9.**	Inhibition of MicroRNA-96 Ameliorates Cognitive Impairment and Inactivation Autophagy Following Chronic Cerebral Hypoperfusion in the Rat	2-VO surgery, Sprague Dawley rats	To identify the effect of a miR-96 on autophagy under CCH	•↑ miR-96 under CCH •Inhibition of miR-96 ameliorated cognitive impairment induced by 2-VO	[135]
**10.**	MicroRNA-9 induces defective trafficking of Nav1.1 and Nav1.2 by targeting Navβ2 protein coding region in rat with chronic brain hypoperfusion	2-VO surgery, Sprague Dawley rats	To investigate the role of miR-9 in regulating Nav1.1/Nav1.2 trafficking in 2-VO model	•↑ miR-9 in both hippocampus and cortex in 2-VO model •miR-9 plays a key role in regulating the process of Nav1.1/Nav1.2 trafficking by targeting Nav2 protein in 2-VO rats	[136]
**11.**	MicroRNA-Mediated Therapy Modulating Blood-Brain Barrier Disruption Improves Vascular Cognitive Impairment	BCAS surgery (0.16mm microcoils), C57BL/6 mice	To elucidate the role of TNFα-responsive miRs in VCI progression	•↑ TNFα gene expression in white matter post-BCAS surgery •↑miR-501-3p by TNFα •TNFα stimulation decreased Claudin-5, ZO-1 and occluding gene expression • TNFα-miR-501-3p-ZO-1 axis plays an important role in pathogenesis of VCI	[137]
**12.**	S-adenosylmethionine Administration Attenuates Low Brain-Derived Neurotrophic Factor Expression Induced by Chronic Cerebrovascular Hypoperfusion or Beta Amyloid Treatment	2-VO surgery, Sprague Dawley rats	To investigate the expression of all BDNF transcripts in the hippocampus with CCH or Aβ injection with SAM treatment	•↓ BDNF mRNAs and protein in hippocampus under CCH and Aβ treatment •SAM improved low BDNF expression through BDNF exons IV and VI	[138]
**13.**	MicroRNA-153 impairs presynaptic plasticity by blocking vesicle release following chronic brain hypoperfusion	2-VO surgery, Sprague Dawley rats	To find out how chronic brain hypoperfusion impairs presynaptic plasticity	•↑miR-153, ↓vesicle fusion-related proteins in 2-VO model • Inhibition of miR-153 ↑ vesicle fusion-related proteins, ↓ cognitive decline in 2-VO model	[176]
**14.**	MicroRNA-195 prevents dendritic degeneration and neuron death in rats following chronic brain hypoperfusion	2-VO surgery, Sprague Dawley rats	To investigate whether miR-195 regulates dendritic morphology and neuronal loss under chronic brain hypoperfusion	•↑miR-195 using lenti-pre-miR-195 prevented dendritic degeneration (total length, numbers and crossings of dendrites) and neuron death (Cleaved caspase 3 and 6 levels)	[177]
**15.**	MicroRNA-27a Promotes Inefficient Lysosomal Clearance in the Hippocampi of Rats Following Chronic Brain Hypoperfusion	2-VO surgery, Sprague Dawley rats	To investigate whether miR-27a is involved in chronic brain hypoperfusion-generated changes in the autophagic-lysosomal system	•↑ miR-27a, ↓ LAMP-2 proteins in hippocampus of 2-VO model resulting in inefficient lysosomal clearance	[178]
**16.**	MiR-9 Regulates the Expression of BACE1 in Dementia Induced by Chronic Brain Hypoperfusion in Rats	2-VO surgery, Sprague Dawley rats	To find out the role of miR-9 in a 2-VO model and the molecular mechanisms underlying its effect	•↑miR-9, ↑BACE1 expression and ↓CREB expression in hippocampus and cortex of 2-VO rats •Inhibition of miR-9 resulted in ↓BACE1 expression and ↑CREB expression	[179]
**17.**	Knockdown of microRNA-195 contributes to protein phosphatase-2A inactivation in rats with chronic brain hypoperfusion	2-VO surgery, Sprague Dawley rats	To investigate the role of miR-195 in regulating PP2A activity following chronic brain hypoperfusion	•↓ miR-195 in chronic blood hypoperfusion involved PP2A inactivity which was mediated by post-transcriptional regulation of PME-1, APP and BACE1 expression	[180]
**18.**	MicroRNA-195 protects against dementia induced by chronic brain hypoperfusion via its anti-amyloidogenic effect in rats	2-VO surgery, Sprague Dawley rats	To study the role of miR-195 in chronic brain hypoperfusion	•↓miR-195 in hippocampus and cortex regions under chronic blood hypoperfusion •miR-195 plays a key role in determining dementia susceptibility in chronic blood hypoperfusion by regulating APP and BACE1 expression at post-transcriptional levels	[181]
**19.**	Activation of Cdk5/p25 and tau phosphorylation following chronic brain hypoperfusion in rats involves microRNA-195 down-regulation	2-VO surgery, Sprague Dawley rats	To investigate whether miR-195 could deregulate amyloid metabolism and tau phosphorylation in chronic brain hypoperfusion	•↓miR-195 in 2-VO rats mediated by Cdk5/p25 activation	[182]
**20.**	Acupuncture Attenuates Inflammation in Microglia of Vascular Dementia Rats by Inhibiting miR-93-Mediated TLR4/MyD88/NF-*κ*B signaling Pathway	2-VO surgery, Wistar rats	To find out whether acupuncture alleviates cognitive impairment by suppressing the miR-93 mediated TLR signalling pathway	•↑miR-93 in 2-VO model •↓ TLR4 by acupuncture, accompanied by ↓miR-93 and MyD88/NF-kB signalling pathway activation, attenuates cognitive impairment associated with inflammation in 2-VO model	[183]
**21.**	Aerobic exercise improves VCI through circRIMS2/miR-186/BDNF-mediated neuronal apoptosis	•Human VCI patient samples •2-VO surgery, C57BL/6 mice	To elucidate the mechanism of aerobic exercise in improving VCI	•↓ circRIMS2, ↓BDNF, ↑miR-186 in serum of VCI patients •↓miR-186, ↑circRIMS2 in 2-VO mice undergoing aerobic exercise	[184]
**22.**	Role of microRNA-126 in vascular cognitive impairment in mice	Multiple microinfarction (MMI) model, C57BL/6 mice	To investigate the role of miR-126 in VaD	•↓ Serum miR-126 expression in MMI mice •Conditional knockout of miR-126 resulted in significant cognitive impairment, decreased CBF, myelin density, axon density, increased inflammation and significant water channel and glymphatic dysfunction	[185]
**23.**	Risk prediction models for dementia constructed by supervised principal component analysis using miRNA expression data	Human patient serum	To explore the role of miRNA as potential biomarkers for different subtypes of dementia using risk prediction models.	•Risk prediction model had an accuracy of 0.873 in a validation cohort in AD using 78 miRNAs, 0.836 with 86miRNAs in VaD validation cohort and 0.825 with 110miRNAs in DLB validation cohort •Study used 1601 Japanese individuals	[186]
**24.**	TDB protects vascular endothelial cells against oxygen-glucose deprivation/reperfusion-induced injury by targeting miR-34a to increase Bcl-2 expression	Oxygen-glucose deprivation/reperfusion insult (Cell culture)	To investigate whether miRNAs can be used as a drug target for treating vascular diseases	•Natural product TDB-induced suppression of miR-34a resulted in ↑Bcl-2 protein, mitochondrial membrane maintenance and survival of vascular cells following OGD/R.	[187]
**25.**	miR-134-5p/Foxp2/Syn1 is involved in cognitive impairment in an early vascular dementia rat model	2-VO surgery, Sprague Dawley rats	To study whether miR-134-5p/Foxp2 contributes to cognitive impairment in an early VaD model	•↑miR-134-5p, ↑cognitive impairment in cortex of VaD model •miR-134-5p was found to target Foxp2 •Silencing of Foxp2 significantly inhibited effect of miR-134-5p on synaptic protein loss	[188]
**26.**	Effect of circular RNA, mmu_circ_0000296, on neuronal apoptosis in chronic cerebral ischaemia via the miR-194-5p/Runx3/Sirt1 axis	•BCAS surgery (Ameroid constrictors), C57BL/6 mice •HT22 cell line under OGD	To study the effect of expression levels of circ_0000296, miR-194-5p, Runx3 in neurons induced with CCI and investigate its interaction with neuronal apoptosis	•↑miR-194-5p, ↓circ_0000296, Runx3, Sirt1 under CCI •Mmu_circ_0000296 plays a key role in regulating neuronal apoptosis induced by CCI through miR-194-5p/Runx3/Sirt1 pathway	[189]
**27.**	Promotive role of microRNA-150 in hippocampal neurons apoptosis in vascular dementia model rats	2-VO surgery, Sprague Dawley rats	To investigate the effect of miR-150 on VaD	•↑miR-150 in VaD model •miR-150 overexpression significantly increased cell apoptosis compared to control •miR-150 antagomiR ameliorated VaD symptoms by upregulating HOXA1 expression	[190]
**28.**	Circulating microRNAs as potential biomarkers for the identification of vascular dementia due to cerebral small vessel disease	Human plasma samples (small vessel VaD patients)	To identify circulating miRNAs in small vessel VaD that holds potential as biomarkers for the disease	•miR-409-3p, miR-502-3p, miR-486-5p and miR-451 as valuable biomarkers •Sensitivity, specificity and AUC for these miRNAs were 76, 75, 75 and 70%; 89, 89, 83 and 75% and 0.94, 0.92, 0.90 and 0.86, respectively.	[191]

Given increasing evidence of miRNAs playing a crucial role in VaD, and its potential in improving disease pathogenesis when modulated, a number of experimental interventions have been explored. Nimodipine, a calcium channel blocker, was observed to attenuate CCH-induced tau phosphorylation via the miR-132/GSK-3β pathway in rat models [136]. Interestingly, acupuncture in rat models of VaD displayed attenuation of inflammation by inhibiting miR-93 that mediates TLR4/MyD88/NF-κB signalling pathway [137]. Aerobic exercise has also been shown to improve VCI through circular RIMS2/miR-186/BDNF-mediated neuronal apoptosis in both serum of VCI patients and in a mouse model of CCH [138]. A recent study by Shi and colleagues has also explored the construction of a competing endogenous RNA (ceRNA), which competes with mRNA for the same pool of miRNAs, immunoregulatory network in relation to VaD through a weighted lncRNA-miRNA-mRNA network analysis [139]. Such research allows identification of new therapeutic targets for VaD in the immunological context.

ncRNAs therefore seem to potentially play an important role in VaD. There is diverse and increasing number of evidence to support this statement. Nevertheless, there is more scope to understand ncRNAs and the various classifications first and then subsequently the role it plays in contributing to the pathogenesis of VaD. Compared to DNA methylation and histone modifications, research on ncRNAs are rather naïve. However, the potential they hold in unravelling the pathogenesis of VaD or as a form of therapeutic intervention should not be dismissed.

## Advancing Translational Epigenetics Therapeutics: Insights from Clinical Trials

The therapeutic potential of epigenetics has been increasingly explored in the realm of clinical trials. For instance, a randomised control trial involved administering inhibitors of epigenetic “readers” aimed at preventing or slowing down dysregulation of gene expression and hence the pathogenesis of the disease. Demented patients, classified based on the Montreal Cognitive Assessment (MoCA) scores, showed improved cognition with the administration of the inhibitor proteins [140]. In another study, intake of folate, vitamin B6 and B12 were significantly associated with better cognitive reserves in mild cognitively impaired patients, and this was implicated to be mediated via DNA methylation [141]. Notably, while the role of epigenetics in the pathogenesis of VaD is pertinent, its therapeutic value in VaD remains poorly explored.

### Therapeutic potential of epigenetics in VaD

Understanding the specific roles of different epigenetic mechanisms not only provides insights into the etiology of VaD but also opens avenues for potential epigenetics-based therapeutic interventions. In the recent years, epigenetic therapeutics has made significant strides, with FDA-approved drugs like Azacitidine and Decitabine (DNMT inhibitors) [142], Vorinostat and Romidepsin (HDAC inhibitors) [143] for treating cancer demonstrating the clinical potential of targeting epigenetic pathways. HDAC inhibitors like RGFP966 are being investigated for AD to enhance cognitive function [144]. However, targeting specific epigenetic mechanisms remains challenging at this stage due to a limited understanding of which mechanisms are the most therapeutically beneficial, as well as a lack of specific molecules capable of effectively targeting these mechanisms. Nevertheless, advancements in our understanding of these mechanisms, coupled with the development of novel drugs targeting epigenetic pathways, hold promise for future therapeutic breakthroughs. While epigenetic therapies hold immense promise, several challenges and limitations must be addressed to fully realize their potential in treating cognitive impairment caused by CCH. A major hurdle is achieving specificity, as epigenetic modifications often affect multiple genes and pathways, potentially leading to off-target effects and unintended consequences, such as the activation of oncogenes or the disruption of normal cellular functions. Additionally, the lack of reliable biomarkers in VCI and VaD complicates patient stratification and the development of personalized treatment approaches. Ethical concerns also arise, particularly regarding the heritable nature of some epigenetic modifications and the potential for unintended generational effects, as well as the use of these therapies in non-life-threatening conditions. However, most VaD patients are beyond the age of reproduction. In addition, although preclinical studies utilizing animal models have offered significant insights into epigenetic mechanisms, their applicability to human clinical contexts remains constrained, and investigations into VaD-specific epigenetics are still at an early stage. Despite these challenges, interdisciplinary collaboration, technological advancements, and careful ethical consideration can help overcome these hurdles, paving the way for transformative epigenetic treatments for VaD and other complex diseases.

Aside from directly targeting components of the epigenetic mechanisms, introducing environmental changes or regimens may also be a possible form of therapeutic intervention given the concept of gene-environmental interactions in epigenetics. A recent study by Belmonte and colleagues reflects the efforts taken to apply this concept in the context of VaD. Repetitive hypoxic preconditioning was used as a form of epigenetic priming to show that the resilience to dementia in mice subjected to CCH was inherited to exhibit intergenerational resilience [145]. While a first of its kind, further experiments are required to validate the strength and applicability of the study as the findings strengthen the relevance of epigenetics-based therapeutic interventions. In fact, being modulators of gene expression patterns, epigenetic mechanisms hold the potential for early detection and preventative treatments as well. Given the contribution of lifestyle-associated risk factors in contributing to VaD, we briefly discuss several lifestyle-associated environmental changes to possibly tackle the challenge at hand.

### Phytochemicals

Phytochemicals refer to plant-derived substances which have the potential to have beneficial effects on human health through their nutritional and medicinal properties [146]. It is interesting to note that phytochemicals are able to mediate these beneficial properties either through specific gene transcriptional activity or via different epigenetic mechanisms. For instance, curcumin, a polyphenol derived from *Curcuma* which is also a substance in turmeric, holds antioxidant and anti-inflammatory properties [147]. It elicits its beneficial effects by regulating epigenetics where it is known to inhibit the activity of both HATs and DNMT1 by blocking its catalytic site through its chemical structure [148]. In addition, anti-ischemic properties of curcumin has been identified for stroke prevention where its effects are postulated to be mediated by epigenetic mechanisms [149]. Resveratrol, a naturally occurring polyphenol in the skin of grapes and berries and peanuts, has been shown to have an epigenetic basis as well [150]. Resveratrol has demonstrated inhibitory effects towards DNMTs and activity against HDAC with a possibility of being a HDAC inhibitor [151]. The neuroprotective effects of resveratrol and its potential in regulating epigenetic changes are thus highlighted. However, its ability to confer neuroprotective effects through modulation of epigenetic mechanisms in neurodegenerative diseases such as VaD has yet to be explored. Specific to VaD, clinical studies on phytochemicals such as rivastigmine and galantamine have proven to be effective in exhibiting beneficial effects in VaD patients [152]. However, further optimisation is required to overcome the adverse side effects.

### Physical Activity & Exercise

It is important to understand the difference between physical activity and exercise as they are often used interchangeably. Physical activity refers to any form of body movement which increases energy expenditure whereas exercise refers to structured and planned activity which aims to improve and maintain physical fitness [153]. Hence, exercise is a subset of physical activity that has been shown to improve motor function and physical strength and prevent or slow down the onset of chronic diseases in human studies [154,155]. It is also suggested to ameliorate cognitive impairment in neurodegenerative animal models [156]. A randomised clinical trial showed early signs of the benefits of aerobic training in improving cognitive functions [157]. The benefits of exercise have long been promoted and even advocated strongly in the public health sector. Epigenetic modifications have been increasingly shown to play a key role in eliciting the beneficial effects of exercise [158]. The effects of parental exercise have also been demonstrated to be inherited by the offspring [154]. The DNA methylome has been identified to be differentially altered in aerobic, anaerobic and resistance training, respectively; while the effects have been noticed to be heterogenous, the involvement of DNA methylation in exercise is affirmed [159]. It is predicted that exercise-induced changes in metabolites affect key enzymes involved in DNA methylation and histone modifications, thus causing epigenetic changes [160]. With respect to neurodegenerative diseases, aerobic or combined strength/aerobic exercise protocols have shown global DNA hypermethylation, hypomethylation of neuroprotective genes such as BDNF and VEGFA and H4 hypoacetylation in leukocytes in animal models and a human study [161]. This serves as validation of physical activity and exercise as a preventative intervention. However, it is important to acknowledge the limitation which lies in the heterogeneity of exercise regimens including the type, intensity and duration which compromises the standardisation in ‘prescribing’ the regimens, and the difficulty in ensuring the intended posture or rigor is reached.

### Dietary Restriction: Intermittent Fasting

Dietary restriction (DR) is generally defined as a voluntary, selected or entire reduction in nutrient consumption without inducing malnutrition [162]. DR comprises mainly of calorie restriction (CR) and intermittent fasting (IF) where it has been demonstrated to be geroprotective by promoting health and longevity [163]. CR refers to an approximately 15-40% reduction in calorie intake [162,164]. On the other hand, IF refers to patterns of eating and fasting (e.g. 16h - 48h) with little or no energy intake [165]. Both CR and IF, individually and in combination, have been shown to have beneficial effects in physiological and pathological conditions. In fact, combining IF with CR has been reported to be effective for increased weight loss and cardio-protection [166]. It is important to note that studies have shown IF to exhibit its beneficial effects by conferring increased resistance to neurons in the brain due to excitotoxic stress independent of CR; and in other cases, IF has been postulated to drive these protective effects even in CR [167,168]. The beneficial effects of IF have even been incorporated in athletes to enhance their performance [169]. IF has been shown to elicit numerous beneficial effects such as reducing body fat, improving insulin sensitivity, reducing inflammation, providing cardio-protective effects, and increasing brain resistance to cardiovascular and neurodegenerative diseases [165]. The role of epigenetics in IF and the mediation of its beneficial effects through the modulation of DNA methylation and histone modifications have also been explored [170]. For instance, a study showed that 36 hours of fasting affected adipose tissue DNA methylation of leptin and adiponectin gene promoters [171]. The inheritability of these epigenetic changes has been explored where it was demonstrated that maternal intermittent fasting prior to mating resulted in modulation of hepatic methylation in the offspring [172]. The role of histone methylation and HDACs have also been implicated with the introduction of IF as well [170]. Apart from the benefits yielded from IF under physiological conditions, its potential is also explored under neurodegenerative diseases. In terms of VaD, IF has been proposed as a promising approach in preventing the disease, although experimental evidence remains slim [173,174,175]. In fact, our group is the first to have studied the effects of 16-hour IF on the DNA methylation landscape in a mouse model of VaD where we showed that IF was able to modulate the DNA methylome to slow down disease progression [77]. There is thus immense potential for IF as a prophylactic intervention to slow down, if not, prevent disease progression of VaD. IF, unlike phytochemical drugs and exercise, is not an introduction of a new element in the lifestyle but rather modulation of an existing eating pattern. This allows scope for standardisation, convenience and ease of compliance, making it a feasible translational intervention. Nevertheless, further experiments are required to validate the findings in VaD patients/at-risk patients and to optimise the IF duration for maximal efficacy.

Furthermore, to broaden the therapeutic scope of epigenetic interventions, it is essential to explore synergies between epigenetic therapies and other complementary approaches, such as neuroprotective strategies and environmental changes. For instance, combining epigenetic drugs with neuroprotective agents (e.g., antioxidants or anti-inflammatory compounds) could enhance their efficacy in mitigating neurodegeneration and promoting neuronal survival. By exploring these synergies, it may be possible to develop more holistic and effective treatment strategies that leverage the interplay between epigenetics, neuroprotection, and lifestyle interventions.

## Conclusion

Epigenetic mechanisms, including DNA methylation, histone modifications, and non-coding RNAs, play a critical role in the pathophysiology of VCI and VaD. Current evidence highlights the significance of gene-environment interactions in VaD, where epigenetic changes act as mediators, transmitting the effects of environmental stressors across generations. These mechanisms provide a novel perspective for understanding the molecular basis of VaD and offer potential targets for therapeutic intervention. Despite advancements, key questions remain unanswered, particularly regarding how epigenetic changes drive vascular dysfunction and CCH. Further research in both human studies and experimental models is essential to delineate these mechanisms and develop effective, pleiotropic interventions. Lifestyle-based strategies, such as phytochemicals, exercise, and intermittent fasting, hold promise due to their ability to modulate epigenetic pathways and address the multifactorial nature of VaD. This review synthesizes current evidence on epigenetic mechanisms in VaD, incorporating studies from various models, regardless of validation status, to provide a comprehensive perspective. However, caution must be exercised when extrapolating findings from animal models to humans. Rigorous validation and critical analysis of these models are necessary to ensure the translatability and safety of epigenetic-based therapies. In conclusion, targeting epigenetic mechanisms offers a transformative approach to understanding and treating VaD. By integrating epigenetic insights with clinical research, we can pave the way for innovative preventive and therapeutic strategies, ultimately improving outcomes for patients with VCI and VaD.
